# Environmental Control on Fish and Macrocrustacean Spring Community-Structure, on an Intertidal Sandy Beach

**DOI:** 10.1371/journal.pone.0117220

**Published:** 2015-01-24

**Authors:** Achwak Benazza, Jonathan Selleslagh, Elsa Breton, Khalef Rabhi, Vincent Cornille, Mahmoud Bacha, Eric Lecuyer, Rachid Amara

**Affiliations:** 1 Université du littoral, UMR 8187 LOG, F-62930 Wimereux, France; 2 CNRS, UMR 8187 LOG, F-62930 Wimereux, France; 3 Université de Bordeaux, CNRS, UMR 5805 EPOC, Station Marine d’Arcachon, place du Docteur Peyneau, F-33120 Arcachon, France; Chinese Academy of Sciences, CHINA

## Abstract

The inter-annual variability of the fish and macrocrustacean spring community on an intertidal sandy beach near the Canche estuary (North of France) was studied from 2000 to 2013 based on weekly spring sampling over an 11-year period. Twenty-eight species representing 21 families were collected during the course of the study. The community was dominated by a few abundant species accounting for > 99% of the total species densities. Most individuals caught were young-of-the-year indicating the importance of this ecosystem for juvenile fishes and macrocrustaceans. Although standard qualitative community ecology metrics (species composition, richness, diversity, evenness and similarity) indicated notable stability over the study period, community structure showed a clear change since 2009. Densities of *P. platessa*, *P. microps* and *A. tobianus* decreased significantly since 2009, whereas over the period 2010-2013, the contribution of *S. sprattus* to total species density increased 4-fold. Co-inertia and generalised linear model analyses identified winter NAO index, water temperature, salinity and suspended particular matter as the major environmental factors explaining these changes. Although the recurrent and dense spring blooms of the Prymnesiophyte *Phaeocystis globosa* is one of the main potential threats in shallow waters of the eastern English Channel, no negative impact of its temporal change was detected on the fish and macrocrustacean spring community structure.

## Introduction

Intertidal ecosystems are dynamic interfaces between the land and the sea. Although these particular environments display harsh and highly variable hydrodynamic conditions, they support a diverse and heterogeneous fauna, and are thought to be highly productive [1,2]. Among intertidal systems worldwide, sandy shorelines are one of the most extensive, dominating most of the temperate coastlines [3]. These ecosystems are important temporary habitats for the life cycle of many marine organisms such as juvenile fish, and consequently, are considered to play an important role for coastal ﬁsheries [4]. Many authors have reported that intertidal areas provide a refuge from predators, abundant food resources and favorable environmental conditions, which promote growth and survival (see [5]).

However, how such unique and vulnerable ecosystems respond to environmental forcing, notably in regards to inter-annual community structure, dynamics and persistence is still poorly understood, particularly for fish and macrocrustacean communities. Indeed, while many marine fish and macrocrustacean species have the ability to use the intertidal zone during high tide, most studies have concentrated on meiofauna and macrobenthos and more recently on birds (e.g. [6]). In addition, the majority of the studies on fish have been conducted on rocky intertidal ecosystems (e.g. [7,8]). This is especially the case for European coasts, where only a few studies have been conducted on exposed sandy beach fish and macrocrustacean communities [2,9,10,11,12]. Furthermore, these studies have mainly been undertaken over a short timescale, no more than 5 years (e.g. [9]). Long-term studies have focused either on population dynamics of one of the dominant species, typically flatfishes [13], or the most abundant motile macrocrustacean groups, in particular crabs and shrimps [14].

Long-term studies are essential to assess the effect of environmental changes and human activities on intertidal fauna communities. Several recent studies revealed changes in coastal sandy shore macrofaunal communities which were directly or indirectly related to long-term climate variability (e.g. [15,16]). Because of its relatively sessile habit, benthic macrofauna is regarded to be a good indicator for environmental changes and disturbances in the marine environment [17] and references therein). However, knowledge on how such environmental changes affect intertidal sandy beach motile fauna, such as fish and macrocrustaceans, is still limited. In the subtidal, many studies have observed and described during the past decade the effects of disturbance and environmental changes on fish, macrocrustacean community structure, and diversity in the English Channel and North Sea (e.g. [18,19,20]). Variations have been related mainly to those of: climate (i.e. the North Atlantic Oscillation (NAO) index), hydrological conditions (temperature and salinity), food, and/or predator abundances. For example, in the eastern English Channel (EEC) hydrological conditions have dramatically changed since the beginning of the 2000s, being influenced by the reduction of freshwater discharge by the main river (the Seine river) [21]. These environmental conditions changes (i.e. lower river discharge and increase of the salinity) have been suggested as the major cause of the suprabenthic faunal changes in the Seine estuary [21]).

Exposed sandy beaches are important habitats along the EEC and southern bight of the North Sea, representing 74% of the mainland coast, and providing important nursery habitat for juvenile fish and macrocrustaceans [2,12,22]. One of the main threats affecting shallow waters of the EEC is the recurrent and dense algal spring bloom of the Prymnesiophyte *Phaeocystis globosa*, which induce foam accumulation on the surface of the sea and beaches by the release of mucilaginous polysaccharides. Colony proliferation affects the penetration of light in the water column, thus seriously impacting on the abundance, metabolism, growth, feeding and behaviour of marine organisms [23]. Both macrobenthic species richness (potential prey for ﬁsh and crustaceans) and densities have been simultaneously reduced during a *Phaeocystis* spring bloom [24]. Although the intertidal zone is potentially the most impacted area by foam accumulation, no inter-annual study to date has analysed its impact on fish and macrocrustaceans.

Based on environmental variables, fish and macrocrustaceans collected during spring (from March to June) over an intermittent 11 year period from 2000 to 2013, this study explored (i) changes in the species composition and community structure of an intertidal sandy beach, and (ii) the underlying environmental factors generating inter-annual variability in fish and macrocrustacean communities, including the potential negative impact of *Phaeocystis* spring blooms.

## Materials and Methods

The permission to collect fish in the areas under study was issued by the “Direction des Affaires Maritmes DAM” of Boulogne-sur-mer (dram-npe@equipement.gouv.fr). In France there is no need for special approval to catch fish by an ethics committee. The present field study did not involve endangered or protected species. This study was conducted in accordance with European Commission recommendation 2007/526/EC, on revised guidelines for the accommodation and care of animals used for experimental and other scientific purposes.

## Study area and sampling

This study was carried out on a sandy beach (Sainte Cécile) located near a small estuary (La Canche), on the French coast of the eastern English Channel. The beach is characterised by the presence of bars and pools parallel to the coast with fine and medium sands. The distance between high and low water marks is about 700 m at neap tide and 1500 m at spring tide. The tidal regime is semi-diurnal with an average tidal range of about 7 m on spring tides and 3 m on neap tides.

For 11 years (2000, 2003–2007, and 2009–2013), sampling was conducted weekly (from March to June) at two replicate stations located at 300–400 m from the high tide line (50° 33’N, 1° 35’E), as soon as the meteorological conditions were favorable. On average 13 sampling dates were done during each spring period except during 2007 where only 8 sampling dates were done. Sampling was done with a 1.5 m beam trawl during daylight hours. The trawl had a 5.5 m long net with a mesh size of 8 x 8 mm in the main body, 5 x 5 mm in the cod end, and was equipped with a tickler-chain in the ground rope. Following recommendations by [25], net speed was kept as constant as possible during sampling, about 38 m min^-1^. The net was pulled by two people in parallel to the shoreline in a water depth <1 m during the ebb tide (high tide +3 h). A meter registered the distance traveled by the trawls. As a result, each trawling represented an average distance of 250 m and a sampling surface of about 400 m^2^. Catches of each trawling were stored in plastic bags and sorted within a few hours in the laboratory.

After the catch, the fish were anesthetized with clove oil and transported in plastic bag to the laboratory. All fish and macrocrustaceans were identified to species level and counted. Individual fish were measured (total length, mm). Small-sized crustaceans (e.g. isopods, mysids) were not included in the present study. For each species, density was calculated as trawl catches standardized to numbers of individuals per 1000 m^-2^ trawled and not corrected for net efficiency. Annual species density was *calculated* as the *average density* of all samples across the spring period (from March to June). Before, species densities obtained from the two sampling stations were averaged at each sampling date.

### Environmental variables

Daily Seine River runoffs at Poses (m^3^ s^-1^) were made freely available by the Public Interest Group Seine-Aval (http://seine-aval.crihan.fr/web/). Data of the NAO winter (December through to March) index (NAO_w_) were obtained from the National Center for Atmospheric Research website (http://www.cgd.ucar.edu/cas/jhurrell/indices.html). NAO_w_ is estimated based on the Sea Level Pressure difference between Lisbon (Portugal) and Stykkisholmur (Iceland) between December and March measured since 1864.

Temperature (T,°C), salinity (S), Suspended Particulate Matter (SPM, mg.l^-1^), chlorophyll-a concentration (Chl-a, µg.l^-1^), and *Phaeocystis globosa* abundance were obtained from the national French monitoring network SOMLIT (http://somlit-db.epoc.u-bordeaux1.fr/download.php?serie=ST). Temperature and salinity were measured with a CTD probe Seabird CTD25 or SBE19. Hydrological data and water samples for phytoplankton counts were collected with 8 L Niskin bottle fortnightly at high tide in subsurface (-2 m) and near the bottom (~ -20 m) from the permanent coastal station C (50°40’75 N; 1°31’17E) located near the study area. For the present study, data from subsurface and near the bottom were averaged at each sampling date.

Chl-*a* was estimated according to the equations of [26], after extraction in acetone 90% for 12h at 4°C in the dark. Abundance of *P*. *globosa* cells was determined under inverted microscopy according to the Utermöhl method from samples preserved with acid Lugol’s iodine solution (2% final concentration) up until 2006, thereafter with 1% (final concentration) Lugol-glutaraldehyde solution. The number of *Phaeocystis* cells of the colonial form was counted separately from free cells within a month after sampling according to biovolume measurements [26], except for samples from the period 2000–2005 which were counted months to several years after sampling. In this case, the number of *Phaeocystis* cells was counted as a total number of free cells, as Lugol’s iodine solution disintegrates the colony matrix some months after fixation [27]. Note that long-term storage in Lugol’s seems do not affect *Phaeocystis* cell abundance. Accordingly, based on cell counts made at 9 sampling dates, a significant relationship (r^2^ = 0.86, p<0.001) with a slope of 1.06 was found between preserved sub-samples with Lugol’s for more one year and those preserved with Lugol-glutaraldehyde counted within a month after sampling (S1 Fig.). *Phaeocystis* biomass was estimated using a mean carbon conversion factor of 89.5 pg C cell^–1^ [26]. *Phaeocystis* spring biomass was calculated by integrating over time the biomass data for the spring bloom period [28]. This choice was motivated by the transient nature of such bloom, and the very high range of biomass values in the course of any spring bloom (up to five orders of magnitude).

### Data analysis

To examine inter-annual variations in environment, a normalized Principal Component Analysis (PCA) was performed to render the environmental variables scale-free and dimensionless [29]. Inter-annual variations in species composition was explored with several indices. Species diversity and evenness were assessed by the Shannon–Wiener and the Pielou’s evenness index, respectively. Similarity in species composition between years was assessed by calculating the Jaccard’s similarity index on presence/absence data for each pair of years [30]. This index ranges from zero (no shared species between years) to one (identical years). In addition, one-way analysis of similarity ANOSIM was performed on species densities to test statistically the variation in spring fish and macrocrustacean assemblages over the 11 years. The test was performed on a Bray-Curtis similarity matrix, calculated using log-transformed data. To examine inter-annual variations in species densities, a centered PCA was chosen to keep variance and dominance among species. Only species with an occurrence >1% in the whole sampling period were considered. To ascertain distinct species assemblages an ascendant hierarchical classification (AHC) with the Ward’s aggregation criterion was built from the first component coordinates from the factorial map of the centered PCA. Temporal trends were tested according to [31]. In the case of autocorrelation in time-series, variance was corrected according to [32].This methodology is described in detail in [33]. Before trend analysis, missing years (2002, 2003, and 2008) were interpolated using distance weighted least squares. Relationships between species densities and environmental variables were explored with two different approaches: (1) a co-inertia analysis was performed with all species for a global measure of co-structure, followed by (2) a generalised linear model (GLM) analysis, both to focus on the dominant species and to determine the relative importance of each significant environmental variable.

Co-inertia analysis consists in finding co-inertia axes, which maximize covariance between row coordinates (years) of environment and species density matrices [34]. Co-inertia was calculated from the normalized PCA on the environmental variables and from the centered PCA on the species densities. The strength of the relationship (i.e. coupling) between these two data-sets was assessed by calculating a multidimensional correlation coefficient (RV) and by testing the statistical significance using the Monte Carlo permutation procedure with 1000 permutations.Generalized linear models (GLM) were applied assuming a Gaussian distribution. Before GLM analysis, multi-collinearity between the environmental variables was tested with Pearson correlation method. Because of collinearity between salinity and Seine river runoff, this last variable was removed from the GLM analysis. The GLM was built with an additive methodology: environmental variables as predictors were tested independently for significance and subsequently, significant ones were added to determine the residual deviance, as well as the percentage explained by each one and the total percentage of the deviance explained by the model. All environmental variable interactions were initially included in the model. The step AIC function (R package “MASS”, v7.3–5) was used to select the significant environmental variables and to estimate the coefficients of the models. Environmental variables were removed by backward elimination based on Akaike’s information criterion (AIC). AIC balanced the degree of fit of a model with the number of variables, in order to find the most parsimonious model. Once the model with the lowest AIC value was selected, the deviance for each of the significant environmental variables was analysed. None of the models revealed any major violation of the modeling assumptions (i.e. residual normality, homoscedasticity). Moreover, inspection of autocorrelation plot of the residuals showed no evidence of autocorrelation outside of the Bartlett two-standard-errors limits.

Kruskal–Wallis tests and Mann–Whitney U test for post hoc pairwise comparisons were performed with XLSTAT 2007 and were used to check for differences in fish species densities between years. ANOSIM analyses were performed with PRIMER software package (version 5.0) [35], PCA and co-inertia analyses with ade4 package and GLM with R software (R Development CoreTeam, 2005), and autocorrelation plot with SYSTAT 11. All statistical significance were set at p <0.05.

## Results

### Environmental variables

**Figure 1:**
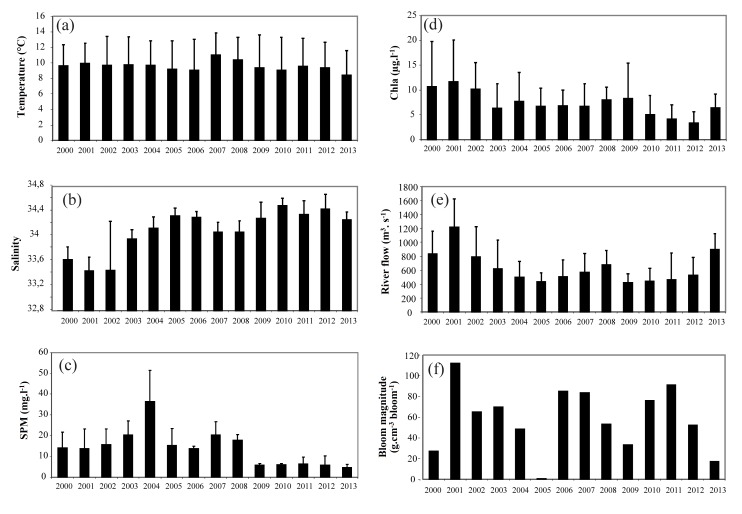
Temporal variations (2000–2013) of environmental variables in coastal waters of the eastern English Channel. (a) temperature, (b) salinity, (c) Suspended Particulate Matter [SPM], (d) chlorophyll-a concentration [Chl-a]), (e) Seine river flow, (f) *Phaeocystis globosa* bloom magnitude.

**Figure 2:**
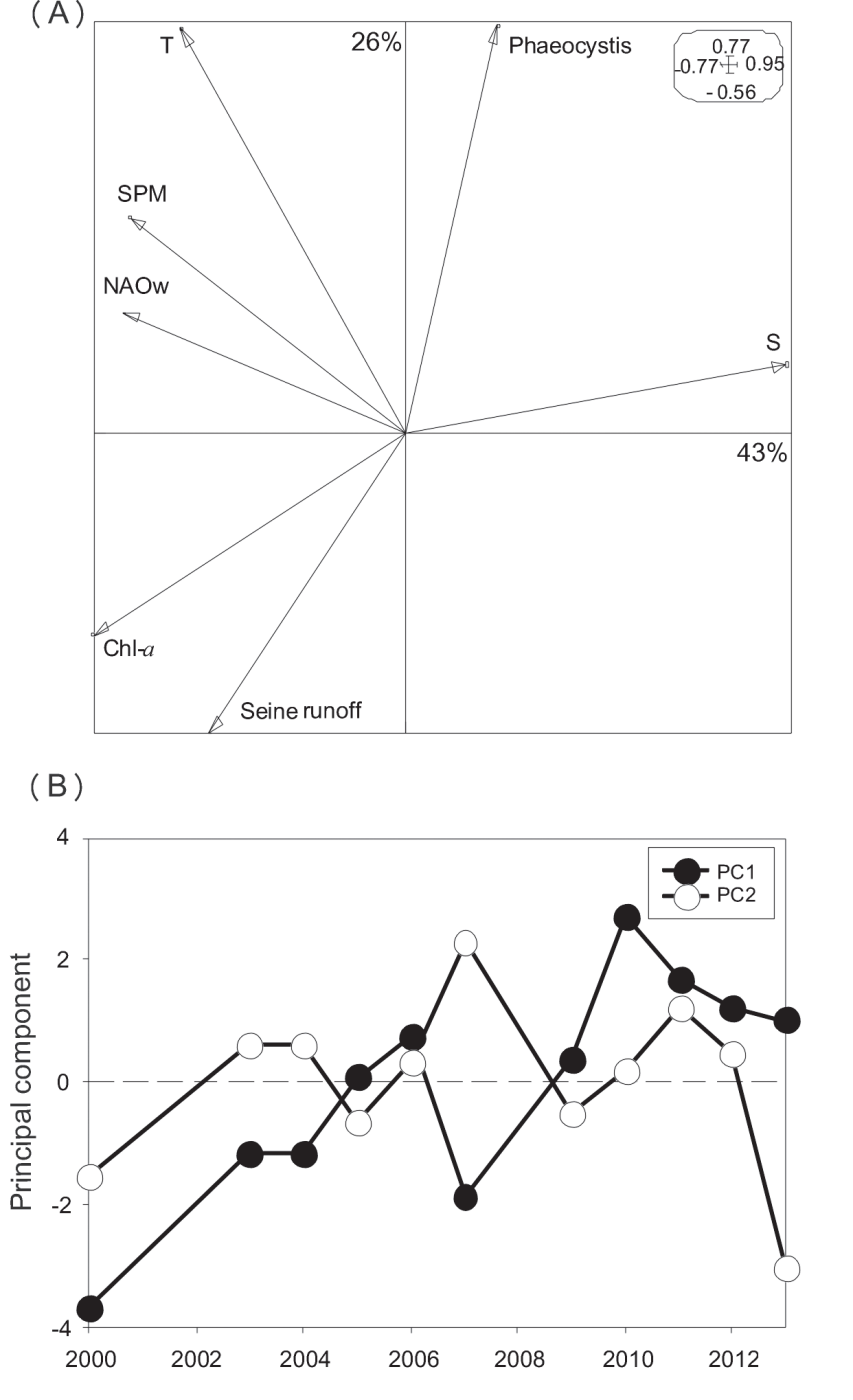
Variations (2000–2013) in climate (NAOw) and environmental variables (Seine River runoff, T, S, SPM, Chl-*a*, and the magnitude of *Phaeocystis* blooms) using a normalised PCA. A. Ordination plot of the variables in the two first principal components (PC1 and PC2). Contribution of each axis to total variance is shown in %. Scales of the axes are given in the boxes. B. Temporal variations in PC1 and PC2. Note that temporal trend in PC1 was highly significant (r^2^ = 0.65, p< 0.001).

All environmental variables during the winter/spring period displayed high inter-annual variations over the period 2000–2013 (S1 Table). For example, mean temperature, salinity, and the magnitude of *Phaeocystis* spring blooms (Fig. 1) varied from 8.5 (2013) to 11.03°C (2007), from 33.9 (2000) to 34.5 (2010), and from 0.29 (2005) to 112 g C m^-3^ bloom^-1^ (2003), respectively. The first and second principal components (PC1 and PC2) of the PCA on all environmental variables explained 43 and 20% of the total data inertia, respectively (Fig. 2). The first principal component (PC1) showed an opposition between salinity and NAOw, Seine River runoff, T, SPM, and Chl-*a* (Fig. 2A). Furthermore, PC1 exhibited a significant increasing temporal trend over the period 2000–2013 (z = 2.29, p< 0.05, Fig. 2B), resulting mainly from the gradual significant increase in salinity (z = 3.18, p< 0.01) over time, but a progressive decrease in both SPM (z = -2.74, p<0.01) and Chl-*a* (z = -2.41, p<0.05). By contrast, no significant temporal trends for NAOw, T, Seine River runoff and *Phaeocystis* were detected (p>0.05).

### Species composition

A total of 28 species (18 fish and 10 macrocrustaceans) belonging to 21 different families, were collected in the intertidal sandy beach over the 11 spring periods (March to June), from 2000–2013 (Table 1). The captured fish were mainly represented by juveniles, often from species of commercial importance. Seven fish species were commonly present each year over the studied period. Among them, *Pleuronectes platessa* (exclusively 0-group), *Sprattus sprattus* (post-larvae and juvenile), and *Pomatoschistus microps*, were the most abundant representing 43.3, 39.2, and 13.5% of the total fish catches, respectively. *Crangon crangon* was by far the most abundant species (mean density during the period of study: 1393 ind. 1000 m^-2^), and contributed to 72% of the total catches. *Carcinus maenas* was also regularly caught (85% frequency of occurrence).

**Table 1:** Species composition, mean densities (ind/1000m²) and occurrence (calculated for the whole study period) of fish and macrocrustaceans along the Sainte Cécile intertidal sandy beach over the period 2000–2013.

Family	Species (abbreviation)		2000	2003	2004	2005	2006	2007	2009	2010	2011	2012	2013	(%) occurrence
Fishes														
Clupeidae	Sprattus sprattus	(S spr)	30.5	212.8	46.5	213	215.1	57	49.2	637	119.8	283	161.8	72.48
	Clupea harengus	(C har)	0	0	0	0	0	0	0	0	0	0.1	0	0.65
Gasterosteidae	Gasterosteus aculeatus	(G acu)	0	0	0	0	0	0	0	0.1	0	0	0	0.65
Gadidae	Merlangius merlangus	(M mer)	0.3	0	0	0	0	0	0	0	0	0	0	1.40
	Trisopterus luscus	(T lus)	0.8	0.3	0	0	0	0	0	0	0	0	0	2.31
Atherinidae	Atherina presbyter	(A pre)	0	0	0	0	0.2	0	0	0	0.2	0.1	0.20	2.92
Agonidae	Agonus cataphractus	(A cat)	1.3	0	0	0	0	0	0	0	0	0	0	2.10
Moronidae	Dicentrarchus labrax	(D lab)	0	0.7	0.3	0.1	0	0	0.5	0.5	0	0.7	1.90	14.85
Gobiidae	Pomatoschistus microps	(P mic)	42.1	243.7	198.2	153	37.4	45.4	3.1	1	1.5	70	6.73	76.29
Cottidae	Cottus gobio	(C gob)	0	0	0	0	0.2	0	0	0	0	0	0	1.30
Trachinidae	Echiichthys vipera	(E vip)	0	0.2	0	0	0	0	0	0	0.1	0	0	1.41
Ammodytidae	Ammodytes tobianus	(A tob)	10.2	25.9	39.5	49.9	26.7	5	2.5	1.2	0.6	0.2	0.59	49.18
Syngnatidae	Syngnathus acus	(S acu)	1	1.4	4.7	0.4	2	0.3	1.2	4.4	4.1	0.3	2.45	31.24
Pleuronectidae	Pleuronectes platessa	(P pla)	509.6	997.2	188.3	294.6	259.7	77.7	63.2	26.5	11.4	69.6	62.16	90.20
	Platichthys flesus	(P fle)	0	0.5	0.4	0	0.1	0	0.3	0.7	0.7	2.1	1.11	19.04
Soleidae	Solea solea	(S sol)	1.2	3.4	2.2	0	0	0	0	0	0	0	0.07	7.13
Scophthalmidae	Psetta maxima	(P max)	0.1	0	0	0	0	0	1	1.7	2.3	1.6	0.49	15.15
	Scophthalmus rhombus	(S rho)	2.3	0	0	0	0	0	0	0	0.9	0.4	0.20	6.93
Macrocrustaceans														
Crangonidae	Crangon crangon	(C cran)	3381.6	756.4	2376.9	1040.9	397.4	847.1	3199.1	431.4	736.3	2260.3	158.92	93.92
Palaemonidae	Palaemon longirostris	(P lon)	0	0.5	0	0.1	0.2	0	0	0	0.1	0.2	0.07	5.40
	Palaemon serratus	(P ser)	0	0	0.4	0	0	0	0	0	0	0	0	1.52
Portunidae	Carcinus maenas	(C mae)	35.3	79.3	78.3	96.3	158.9	63.9	14.4	11.9	6.8	15.2	17.03	84.91
	Liocarcinus holsatus	(L hol)	1.9	0	0	0	0.1	10.5	0	0.1	0	0	0	8.52
Carcinidae	Portumnus latipes	(P lat)	0	2.7	3.9	2.1	0.7	1.2	2.1	0	23.9	2	3.01	30.94
Majidae	Macropodia longirostris	(M lon)	0.3	0	0.3	0	0.1	0	0	0	0	0	0	2.86
Porcellanidae	Pisidia longicornis	(Pi lon)	0.7	2	1	0	0	0	1.3	0	0.2	0	0.20	5.26
Varunidae	Eriocheir sinensis	(E sin)	0	0	0	0	0	0	0	0	0.1	0	0	0.51
	Hemigrapsus sanguineus	(H san)	0	0	0	0	0	0	0	0	0.2	0	0.20	0.98
Total species number			16	15	14	10	14	9	12	12	17	15	17	

Note the absence of sampling in the years 2001–2002 and 2008.

Out of the 28 species captured, only six (*P*. *platessa*, *P*. *microps*, *S*. *sprattus*, *Ammodytes tobianus*, *C*. *crangon*, and *C*. *maenas*) dominated the intertidal zone assemblages over the 11 years, and so could be considered as key species. Accordingly, these species represented in total 99.4% of the total catches, having relative high densities and occurrence during the 11 years studied (Table 1). Average total density of these key species was 1932 ind. 1000 m^-2^. By contrast, most of the other species occurred in low densities or occasionally, such as *Dicentrarchus labrax*, *Syngnatus acus*, *Psetta maxima*, and *Portumnus latipes*. It should be noted that *Eriocheir sinensis* and *Hemigrapsus sanguineus*, two east-Asian introduced species, were recorded in the intertidal zone from 2011.

### Inter-annual variations in community structure and species density

**Figure 3:**
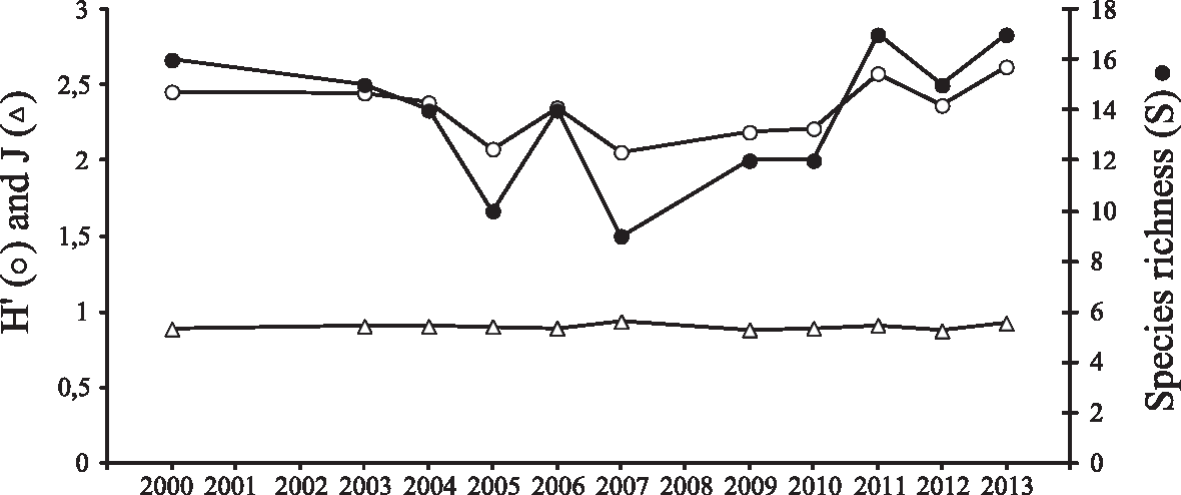
Temporal variations (2000–2013) in species richness (S), diversity (H’) and evenness (J) of fish and macrocrustaceans along the Sainte Cécile intertidal sandy beach. Note the absence of sampling in the years 2001–2002, and 2008.

The various diversity indices (species richness S, Shannon-Wiener diversity H’ and Pielou’s evenness J) exhibited no significant trend (r^2^<0.02; p>0.05) (Fig. 3) and species composition stayed rather similar between years. Among years comparisons exhibited high Jaccard’s coefficients (0.40–0.83) showing that the fish and macrocrustacean species composition was more or less stable between years in a qualitative point of view. Accordingly, only some occasional species (*P*. *flesus*, *D*. *labrax and P*. *maxima)* occurred more frequently from 2009.

**Figure 4:**
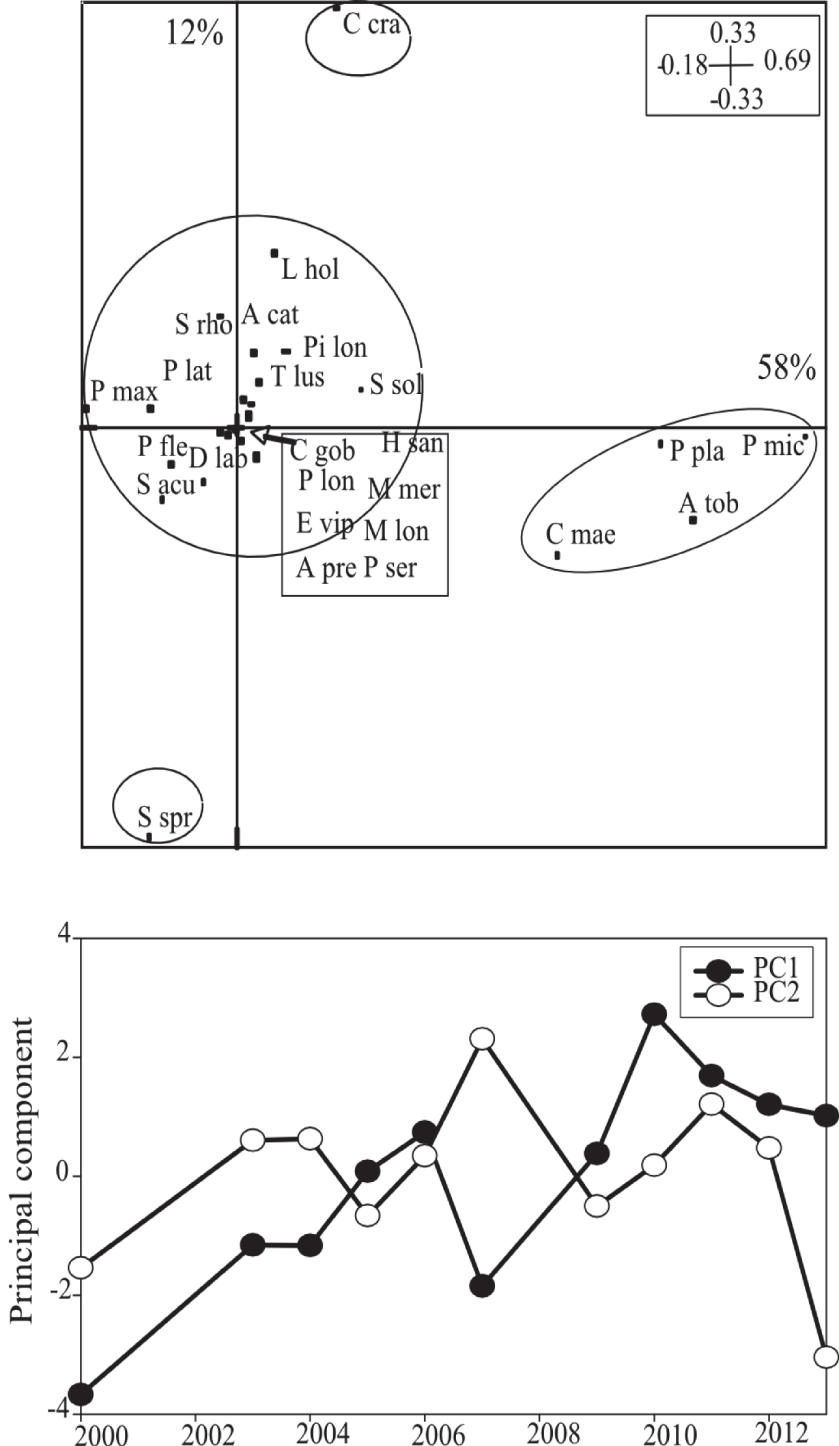
Temporal variations (2000–2013) in fish and macrocrustacean density along the Sainte Cécile intertidal sandy beach using a centred PCA. A. Average position of the species in the two first principal components (PC1 and PC2). Contribution of each axis to total variance is shown in %. Scales of the axes are given in the boxes. Species associations were defined from hierarchical cluster analysis by Ward’s method of the species coordinates. See Table 1 for species labels. B. Temporal variations in PC1 and PC2. Note that temporal trend in PC1 was highly significant (r^2^ = 0.68, p < 0.005), and the absence of sampling in the years 2001–2002 and 2008.

**Figure 5:**
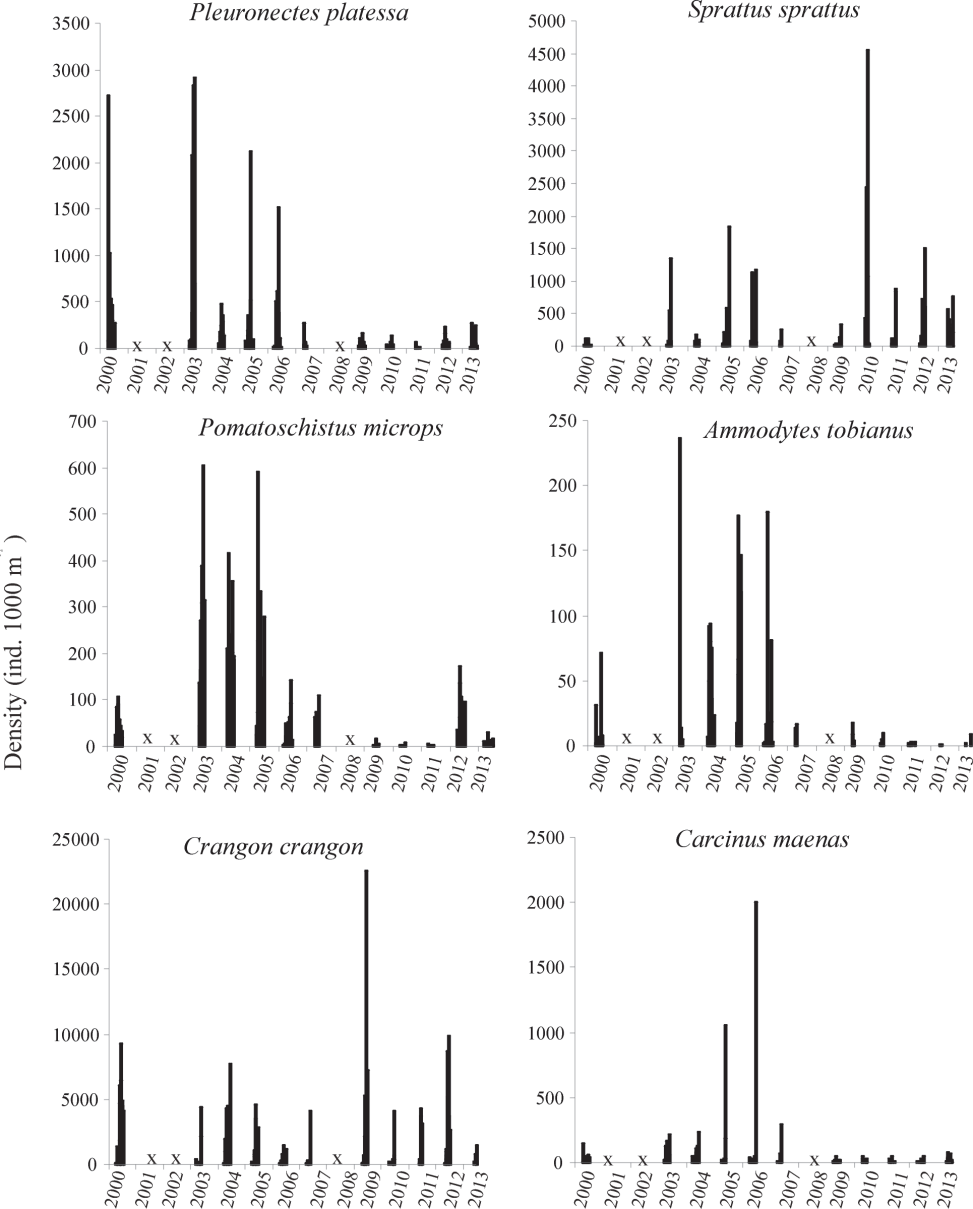
Temporal variations (2000–2013) in densities (ind. 1000 m^-2^) of the dominant fish and macrocrustacean species along the Sainte Cécile intertidal sandy beach. Each bar represent the density at a sampling date. “X” indicate years with no sampling (2001–2002, and 2008). Note the differences in the y-axis scales.

**Figure 6:**
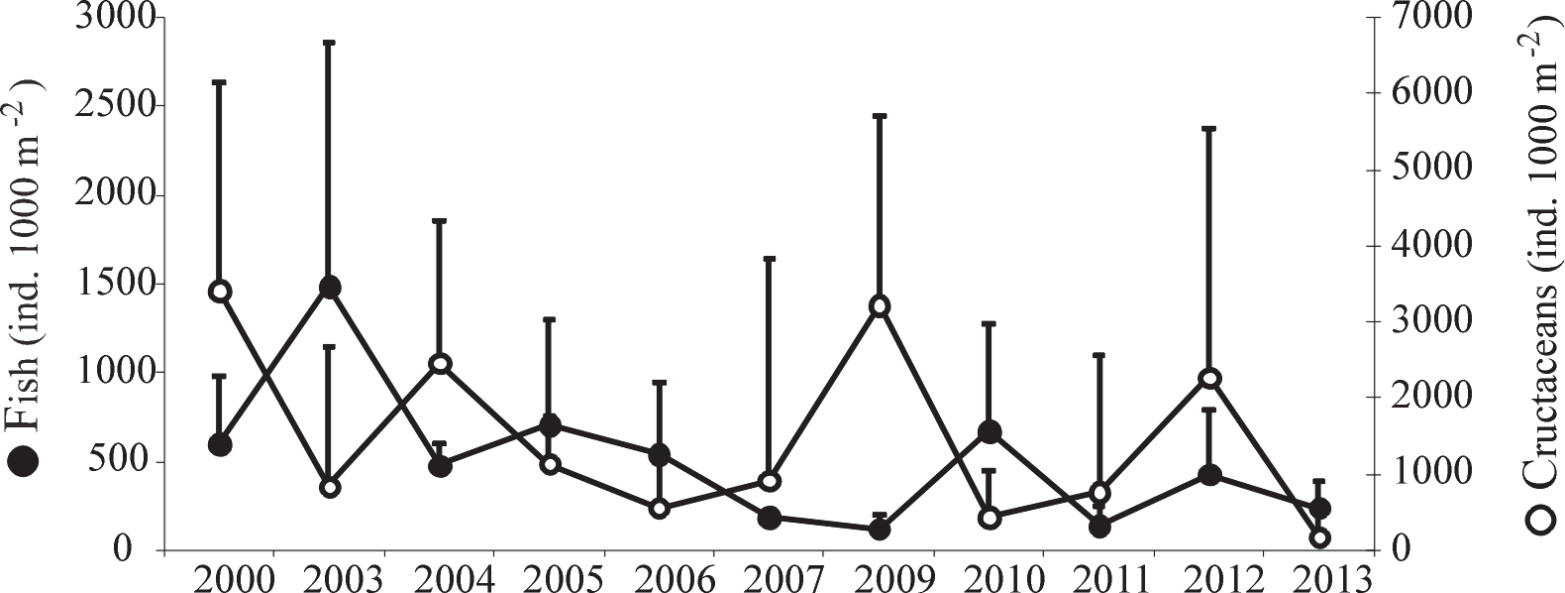
Temporal variations (2000–2013) in mean (+SD) total fish and macrocrustacean densities (ind. 1000 m^-2^) along the Sainte Cécile intertidal sandy beach. Note the absence of sampling in the years 2001–2002, and 2008.

All of the species presented high inter-annual variability in density. One-way ANOSIM indicated a significant change of fish and macrocrustacean assemblages over the 11 years (ANOSIM, *global R* = 0.40, *p* = 0.001). PCA analysis on species density revealed four species assemblages over the 11 years studied (Fig. 4A). The first species assemblage positioned along the first principal component (PC1, 58% of total variance) was composed of *P*. *platessa*, *P*. *microps*, *A*. *tobianus* and *C*. *maenas*. The second and third assemblages in opposition along the second principal component (PC2, 12% of total variance) were composed of *S*. *sprattus* and *C*. *crangon*, respectively. Density of each of these six dominant species (key species) exhibited significant differences between years (Kruskal-Wallis, p<0.05, Fig. 5). Furthermore, PC1 exhibited a significant negative temporal trend (z = -2.96, p<0.01), showing that *P*. *platessa*, *P*. *microps*, *A*. *tobianus*, and *C*. *maenas* progressively declined over the eleven years period. Although PC2 did not display any significant temporal trend, the contribution of *S*. *sprattus* in the catch increased from 2010 (Mann-Whitney test, p<0.05, Fig. 5), contributing to 30±22% of the total catches over the period 2010–2013 against 7±7% previous to this. By contrast, neither *C*. *crangon* nor the fourth assemblage, which was composed of all the 19 other species (Fig. 5A), displayed any significant temporal trend. Altogether, these results showed that the period 2000–2007 was characterized by relative high densities of *P*. *platessa*, *P*. *microps*, *A*. *tobianus*, and *C*. *maenas* and by a low contribution of *S*. *sprattus* within the community. By contrast, the period 2010–2013, was characterized by a relatively high contribution of *S*. *sprattus*, but relatively low densities of *P*. *platessa*, *P*. *microps*, *A*. *tobianus*, and *C*. *maenas*. Note that although fish and macrocrustacean densities fluctuated inversely from year-to-year over the 11 years period (Fig. 6), the negative relationship was not significant (r = 0.15; p = 0. 651).

### Relationships between fish and macroscrustacean spring community-structure, and their environment

**Figure 7:**
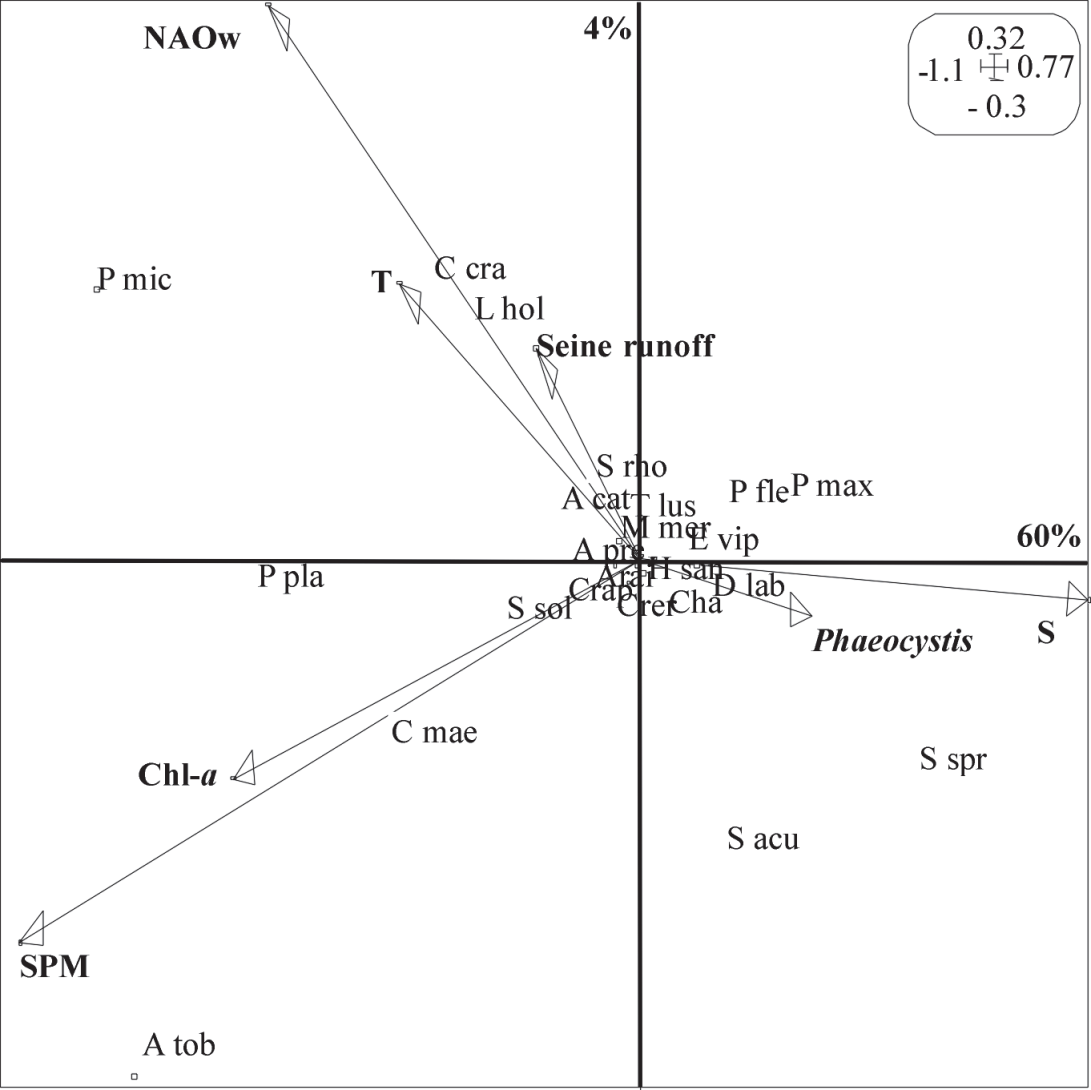
Relationships over the period (2000–2013) between fish and macroscrustacean densities and climate and environmental parameters using a co-inertia analysis. Contribution of each axis to total variance explained is shown in %. Scales of the axes are given in the boxes. See Table 1 for species labels. Note the absence of fish and macrocrustacean species sampling in the years 2001–2002 and 2008.

Co-inertia analysis (Fig. 7) revealed a significant coupling between fish and macrocrustacean spring community-structure, densities and environment (RV = 0.59, p<0.05). Accordingly, the first axis was clearly dominant, and explained alone 60% of the total variance. Co-inertia and GLM results indicated that out of the seven environmental variables, NAOw, T, S, Chl-*a*, and SPM were the most important for structuring the inter-annual distribution of fish and macrocrustacean spring community (Fig. 7, Table 2). Species such as *P*. *platessa*, *P*. *microps*, *A*. *tobianus*, *C*. *crangon* and *C*. *maenas* were relatively more abundant during periods of relative high NAOw, T, Chl-*a*, and SPM. *P*. *platessa* and *A*. *tobianus* densities were negatively correlated to S, while *S*. *sprattus* was negatively correlated to T and Chl*-a*. All models explained substantial proportions of the variance in time series (Table 2). The magnitude of the *Phaeocystis spring blooms was* not well represented on the factorial map of the co-inertia analysis and was not a significant predictor in the GLM analysis, indicating its relative lack of importance in explaining intertidal fish and macrocrustacean spring community-structure.

**Table 2:** Deviance analysis table of explanatory variables in the Gaussian GLM model for *S*. *sprattus*, *P*. *platessa*, *P*. *microps*, *A*. *tobianus*, *C*. *crangon* and *C*. *maenas* densities.

Source of variation		Deviance	Residual dev.	Change in dev.	% Explained	AIC	*p*-Value
S. sprattus	NULL		1,64				
	T	0,37	1,27	0,37	23,78		0,044
	Chla	0,76	0,52	0,76	49,22		0,009
	Total model				73,00	5,57	
P. platessa	NULL		3,27				
	S	1,52	1,72	1,55	47,10		0,011
	SPM	0,88	0,70	1,02	30,90		0,034
	Total model				78,00	12,93	
P. microps	NULL		7,40				
	NAO	2,11	4,64	2,76	37,87		0,005
	SPM	3,91	0,69	3,95	54,13		0,001
	Total model				92,00	12,77	
A. tobianus	NULL		6,90				
	S	1,15	5,48	2,36	18,04		0,022
	Chla	0,95	4,54	4,76	36,33		0,031
	SPM	3,81	0,73	4,54	34,63		0,001
	Total model				89,00	13,31	
C. crangon	NULL		1,79				
	NAO	0,97	0,83	0,97	24,36		0,010
	Total model				66,00	8,72	
C. maenas	NULL		2,04				
	SPM	0,99	1,05	0,99	0,18		0,017
	Total model				0,56	11,41	

Values of deviance for each factor, residual deviance (res. dev.), change in deviance, percentage of the total deviance explained by each factor (% Explained), total model (in bold), and p values are presented.

## Discussion

### Species composition and community structure

Fish and macrocrustaceans are important components of sandy beach fauna. This study found that the great majority of fish and macrocrustaceans were juvenile migrants. The advantages for marine species using the intertidal zone during part of their life cycle are not well known. Many authors have reported that intertidal sandy beach areas may provide refuge from predation coupled with high productivity to enhance survival and growth [36,37]. Species may also use these habitats in search of an optimum physiological environment that promotes maximal growth [38]. Juveniles of some abundant species in the studied area, such as *Solea solea*, *Limanda limanda*, *Buglossidium lutheum* and *Callionymus lyra* [22], remain during spring or summer in the subtidal zone, and are scarcely ever caught intertidally. These species are probably less adapted to fluctuating environmental conditions with their resultant physiological demands. Therefore, utilization of the intertidal zone by certain fish species represents an advantage that not only reduces predation and maximises growth but also reduces competition for food in the earliest stages of their life cycle when densities are highest and competition is likely to be greatest in the subtidal zone [36,39].

The species diversity in this studied intertidal zone was similar to that found in the few other published studies undertaken on European intertidal sandy beaches (e.g. 20 species in a Belgian surf zone [2], and 24 species at Porto Pim, Azores [10]. However, 43 species of fish and 16 species of macrocrustaceans were caught during a four year study on a Scottish intertidal sandy beach [9], and 35 species were captured on other Scottish beaches [40]. Although caution is needed when comparing studies in which different types of fishing gear (different type, size or speed) were used, the beam trawl used in this work is considered efficient for catching demersal fish and epibenthic species in shallow coastal waters [41].

As for other sandy beaches, only few taxa numerically dominated the catch, with most species occurring occasionally or rarely, in low number [2,9,42]. Out of the 28 species captured, only six (*P*. *platessa*, *P*. *microps*, *A*. *tobianus*, *S*. *sprattus*, *C*. *crangon* and *C*. *maenas*) could be considered as dominant and key species of the intertidal zone. They represented 99.4% of the total catches during spring, and showed high densities and occurrence during the 11 years studied. Among them, *P*. *platessa* was the most abundant fish species (43.3% of the total fish catches). As in other shallow waters in northern Europe, *C*. *crangon* (96% of the total crustaceans) was the dominant mobile epibenthic species. This macrocrustacean species is highly abundant and an ubiquitous member of the large motile epifauna of shallow soft bottom areas along the European coasts [2,9,14,43]. The temporal structure of epibenthic communities of temperate intertidal sandy beaches is often the result of seasonal settling or consecutive migration waves of the young stages [11,37]. Indeed, most of the species spawn in deeper offshore waters, and invade shallow coastal areas in spring or summer as late larvae or early juveniles, when such areas are relatively warm and rich in food [10,22,44]. According to [45], a habitat can be defined as a nursery of a species if its contribution per area to the production of recruits to the adult population is greater, on average, than the production from all other juvenile habitats. The juvenile density of most of the key species (e.g *P*. *platessa* 215 ind. 1000 m^-2^) in this study’s intertidal zone was higher than in the adjacent subtidal area (e.g. *P*. *platessa* 16.5 ind. 1000 m^-2^; [22], and in the Canche estuary (e.g. *P*. *platessa* 17 ind. 1000 m^-2^, [46]). In the Dutch Wadden Sea, [47] observed differences between intertidal and subtidal 0-group plaice growth. These authors showed that growth in the intertidal zone was always higher than in the subtidal one, due to the presence of the prey *Arenicola marina* in the intertidal zone. All these data emphasize the importance of sandy intertidal zone as nursery ground, which allows suitable conditions for juvenile fish development.

### Temporal community structure evolution and environmental influence

The fish and macrocrustacean spring community structure in the Canche intertidal zone was stable from year-to-year, and was based on a set of regularly occurring species. Nevertheless, some occasional species (*P*. *flesus*, *D*. *labrax*, *P*. *maxima)* occurred in this study more frequently from 2009. Moreover, some species such as *E*. *sinensis* and *H*. *sanguineus*, which are native to the east coast of Asia, and were introduced into the English Channel during the mid 1930s, and the North Sea during the 1990s [48], were recorded from 2011. Even if these two species are classified as invasive species, they were in low numbers at the beach sampled in our study.

Between years comparisons exhibited high Jaccard’s similarity coefficients (0.40–0.83) and the ANOSIM (based on species densities) showed that the fish and macrocrustacean composition was stable over the 11 years studied. These Jaccard coefficient values were similar to those found on the west coast of Scotland (0.55 to 0.71, [9]), the Belgian surf zone in (0.3 to 0.8, [2]), and Maine, USA (0.38 to 0.83, [42]). Although all these other studies were undertaken over a shorter time scale (less than 5 years), they indicate that the assemblages of intertidal fish are persistent and resilient to any change, and, therefore, their general taxonomic structure is predictable from year-to-year [5,49]. In temperate intertidal ecosystems, the major temporal differences in community structure generally occur on a seasonal basis and reflect the breeding cycle of the species [9,12,44].

Gross temporal patterns of change in fish and macrocrustacean assemblage are mainly a result of alterations in the density of the dominant species. High inter-annual variations in density of the key species were associated to low inter-annual variations in species composition. This is typical of many intertidal fish assemblages [10,42,50] and particularly those inhabiting the rocky intertidal zone (see the review of [5]). Although temporal variations in shallow coastal epibenthic species densities are well documented, much confusion still prevails over the factors influencing or controlling them. They have been attributed to fluctuations in a wide variety of both biological and physical variables including the timing of spawning seasons and hence the influx (immigration/recruitment) and efflux (emigration) of individuals to and from populations (e.g. [9,51]), food availability [52], predation pressure [53,54], water temperature (e.g. [2,55]), wind speed and direction (e.g. [9,54]), turbidity (e.g. [50]), and salinity (e.g. [14]).

Inter-annual variations observed in species density (up by five-fold for the key species), are most likely the result of differences in year-class strength and recruitment success. The fact that fluctuations did not follow the same pattern for all dominant species, suggests that species responded differently to the environmental variables and that the factors contributing to successful recruitment differed between species. It is now clear that climate variability affects the density and biogeography of marine organisms [56]. Several recent long-term studies in the English Channel and the North Sea have revealed changes in intertidal beach macrofauna that were directly or indirectly related to climate variability [15,16]. A study based on a 34-year time series of *C*. *crangon* abundances in the Dutch Wadden Sea indicated that salinity, freshwater discharge and the NAO were relevant factors affecting its abundance [14]. The NAO and river flow may influence recruitment success, probably due to their effects on the productivity and growth of coastal organisms [57]. Such environmental factors have been recognized as key issues in the estuarine colonization and settlement processes of both marine fish and invertebrate larvae and juveniles (e.g. [58,59]).

Co-inertia and generalised linear model analyses identified, among the measured environmental variables, winter NAO index, water temperature, salinity suspended particular matter, and chlorophyll-*a* as the major environmental factors to explain these changes. Hydrological and oceanographic features in coastal waters of the EEC depend mainly on the Seine River outflow, which creates a water mass of relatively low salinity and nutrient enrichment known as “*Fleuve côtier”* [60], spreading northward parallel to the French coast.. Since this frontal zone has been shown to influence fluctuations of ichthyoplankton assemblages such as offshore/onshore distribution [61], the observed hydrological change over the period 2000–2013, associated with NAO change, may have affected progressively the recruitment success of species such as *P*. *platessa*, *P*. *microps*, and *A*. *tobianus*. Density of *S*. *sprattus*, considered as a cold/temperate water species, was negatively correlated to temperature in our study. This species may have probably found optimal conditions for its development when water temperature decreases. [59] illustrated a situation in which climatic oscillations exerted effects on fish assemblages by affecting the suitability of estuarine nursery grounds for marine fish. They found an increase in diversity during high NAO winters, which is partly explained by the increase in the number of rare species. Although species diversity and community structure in our intertidal zone was stable from year-to-year, the NAO was identified as a factor structuring the inter-annual variations in density of some key species such as *P*. *microps and C*. *crangon*. The NAO is probably best viewed as a means of describing processes that operate at a higher level of control of oceanographic and atmospheric phenomena rather than as an agent that acts directly on the fish assemblages.

In shallow coastal areas, juvenile fish are exposed to high predation pressure [58,62]. During the 11 years studied, fish and macrocrustacean densities fluctuated from year-to-year, with a slight negative, but not significative correlation. Many studies have showed predator-prey interactions between these two groups of species. Due to its high density (72% of the total catches in the present study), *C*. *crangon* is a key component of the trophic web. It is known as an important prey for some fishes (gobies, gadoids, several flatfish and demersal roundfish species), crustaceans and shorebirds [14] as well as an important predator of numerous larvae and juvenile stages of several fish and benthic species [53,63]. For example, both *C*. *crangon* and *C*. *maenas* are the main predators on the early benthic stages of *P*. *platessa* in spring [53].

Finally, despite high inter-annual variations in the magnitude of *P*. *globosa* spring blooms, by two-orders of magnitude, no effects on either juvenile fish or macrocrustacean species diversity, densities or assemblages were observed. These results concur with those from a recent mesocosm study, which clearly showed that exudates and TEP excreted from decaying *P*. *globosa* colonies and foam accumulation have no negative effect on juvenile sea bass growth, condition or survival [64]. By contrast to macrobenthic species [24] which can be affected by foam accumulation, fish and macrocrustaceans are motile species, probably giving them the possibilities to avoid foam accumulation on the beach, and unfavourable areas in general.

In conclusion, the fish and macrocrustacean spring community structure in the studied intertidal zone was stable from year-to-year, and was based on a set of regularly occurring species. However, strong inter-annual variations in species density occurred (up by five-fold for the key species), being associated to inter-annual variations in winter NAO index, water temperature, salinity, suspended particular matter and chlorophyll-*a*. No significant relationship between crustaceans and fish were found in the present study but predator-prey interactions should be investigated in more details in future studies. Although the recurrent and dense spring blooms of the Prymnesiophyte *Phaeocystis globosa* is one of the main potential threats in shallow waters of the eastern English Channel, no negative impact of its temporal change was detected on the fish and macrocrustacean spring community structure. More work is necessary both to characterise patterns of faunal change and their linkages with biological and oceanographic changes, and also to understand the causal mechanisms through which fish assemblages are being affected by changing environmental conditions.

## Supporting Information

S1 Fig Comparison of *Phaeocystis* cell counts in replication samples preserved with Lugol’s for more one year and Lugol-glutaraldehyde preserved less than one month after sampling. (TIF)

S1 Table Mean values of environmental data recorded during the first six months (January to June) from 2000 to 2013. (XLSX)
