# Correction: Environmental Control on Fish and Macrocrustacean Spring Community-Structure, on an Intertidal Sandy Beach

**DOI:** 10.1371/journal.pone.0128961

**Published:** 2015-06-29

**Authors:** Achwak Benazza, Jonathan Selleslagh, Elsa Breton, Khalef Rabhi, Vincent Cornille, Mahmoud Bacha, Eric Lecuyer, Rachid Amara

There are errors in the author affiliations. The affiliations should appear as shown here:

Achwak Benazza^1,2^, Jonathan Selleslagh^1,3^, Elsa Breton^1^, Khalef Rabhi^1^, Vincent Cornille^1^, Mahmoud Bacha^1^, Eric Lecuyer^4^, Rachid Amara^1^

1 Université du littoral, UMR 8187 LOG, F-62930 Wimereux, France, 2 Université Badji Mokhtar, Laboratoire d’Ecobiologie Milieux Marins et Littoraux, Annaba, Algeria, 3 Université de Bordeaux, CNRS, UMR 5805 EPOC, Station Marine d’Arcachon, place du Docteur Peyneau, F-33120 Arcachon, France, 4 CNRS, UMR 8187 LOG, F-62930 Wimereux, France
