# Supplementary figures and images for: Environmental Control on Fish and Macrocrustacean Spring Community-Structure, on an Intertidal Sandy Beach
Source: PLoS One. 2015 Jan 24;10(1):e0117220. doi: 10.1371/journal.pone.0117220 (PMC4305308; doi:10.1371/journal.pone.0117220)

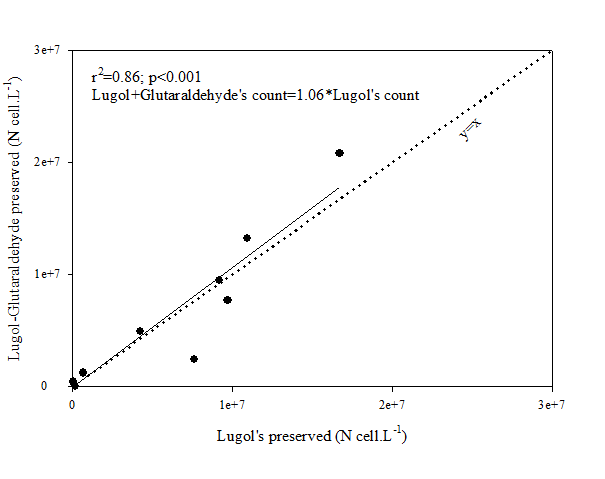

Supplement: S1 Fig — (TIF) [file pone.0117220.s001.tif]
